# Decreased Blood Asprosin in Hyperglycemic Menopausal Women as a Result of Whole-Body Cryotherapy Regardless of Metabolic Syndrome

**DOI:** 10.3390/jcm8091428

**Published:** 2019-09-10

**Authors:** Magdalena Wiecek, Jadwiga Szymura, Justyna Sproull, Zbigniew Szygula

**Affiliations:** 1 Department of Physiology and Biochemistry, Faculty of Physical Education and Sport, University of Physical Education in Krakow, 31-571 Krakow, Poland; 2 Department of Clinical Rehabilitation, Faculty of Motor Rehabilitation, University of Physical Education in Krakow, 31-571 Krakow, Poland; 3 Faculty of Physical Education and Sport, University of Physical Education in Krakow, 31-571 Krakow, Poland; 4 Institute of Health Sciences, State Higher Vocational School in Tarnow, 33-100 Tarnow, Poland

**Keywords:** whole-body cryotherapy, asprosin, metabolic syndrome, hyperglycemia, adipocytokines, menopause

## Abstract

Endocrine dysfunction often occurs in metabolic syndrome (MetS), resulting in hyperglycemia and atherogenic blood lipid profile disorders. Asprosin is a newly discovered glucose-regulating hormone. The study aim was to determine whether the application of whole-body cryotherapy (WBC) affects asprosin and selected adipocytokines as well as insulin resistance in menopausal women with metabolic disorders. A total of 37 menopausal women were exposed to 20 WBC (−130 °C, 3 min). Blood glucose, asprosin, irisin, leptin, adiponectin, and insulin were measured before and after 20 WBC treatments, after which a homeostasis model assessment of insulin resistance (HOMA-IR) and atherogenic index of plasma (AIP) were calculated. The results were analyzed in the MetS group compared to the controls (CON) without MetS, and in the hyperglycemic (HG) group compared to the normoglycemic group (NG). After 20 WBC, a significant reduction (*p* < 0.05) in asprosin concentration was found in the MetS, HG, and CON groups, and a significant decrease (*p* < 0.05) in glucose concentration was noted in the HG group. Changes in asprosin concentration positively correlated with changes in glucose concentration. Asprosin concentration before WBC correlated positively with metabolic disorder risk factor levels, and the change in asprosin concentration after 20 WBC correlated negatively with metabolic disorder risk factor levels: fasting glucose, AIP, and the leptin/adiponectin index. Research indicates the possibility of using WBC in supporting metabolic disorders, type 2 diabetes (T2DM), and insulin resistance.

## 1. Introduction

Metabolic syndrome (MetS) is a set of coexisting disorders that consist of elevated blood pressure, hyperglycemia, and atherogenic disorders of blood lipid profile [1,2,3]. These disorders increase the risk of developing cardiovascular disease (CVD), insulin resistance, and type 2 diabetes (T2DM) [1,2,3,4,5,6]. Visceral obesity is the most common disorder in MetS [7].

MetS was found in 34.7% of the general adult population in the United States; however, regardless of gender, the incidence of MetS increased with age: 18.3% among people aged 20–39, 35% among those aged 40–59 years, and 46.7% among people 60 and above [4]. In older individuals (≥60 years), MetS was more common in women than in men [4,8]. In the Central European region, the incidence of MetS in women aged 60–74 was 46.3%, and was about 12% higher than in men of the same age [8].

In MetS, carbohydrate and lipid metabolism disorders are associated with the dysfunction of adipocyte endocrine function (elevated leptin levels, decreased levels of adiponectin and irisin) and pancreatic β cells and/or tissue insensitivity to these hormones [9,10,11]. The ratio of leptin and adiponectin concentrations, which correlates positively with the homeostasis model assessment of insulin resistance (HOMA-IR), may be an indicator of the development of metabolic complications [12]. It has been demonstrated that an increase in the concentration of irisin causes browning of the white adipose tissue, which is connected with an increase in the expression of uncoupling protein 1 (UCP-1) [13]. Irisin has a beneficial effect on glucose homeostasis and insulin sensitivity by increasing energy expenditure, enhancing glycogenolysis and glycolysis, as well as decreasing gluconeogenesis, adipogenesis, and lipid accumulation [14,15,16]. The level of irisin in the blood correlates positively with the concentration of adiponectin [17] and negatively with leptin concentration [18]. These were statistically significant correlations [17,18].

Asprosin is a recently discovered peptide hormone of the white adipose tissue (C-terminal cleavage product of profibrillin encoded by *FBN1*), with peak fasting secretion regulating the release of glucose from hepatocytes via the G-protein/cAMP-protein kinase A pathway [19]. Its function is also associated with adipogenesis [20]. It is postulated that asprosin crosses the blood–brain barrier and acts orexigenously directly by activating AgRP^+^ neurons via the cAMP-dependent pathway [21]. Higher concentration levels of circulating asprosin have been reported in obese adults and in those with T2DM, as well as in women with polycystic ovary syndrome (PCOS), which occurs in MetS [19,22,23,24]. However, in obese children, the concentration of asprosin was lower than in children with normal body mass [25]. There were positive (statistically significant) correlations between the concentration of asprosin and the concentration of fasting glucose, HOMA-IR, triglycerides (TG), body mass index (BMI), and waist–hip ratio (WHR) in people with T2DM [22]. In women with PCOS, there were significant positive correlations between asprosin concentration and BMI, and fasting insulin and glucose concentrations, HOMA-IR, and C-reactive protein (CRP) [23]. Research by Jung et al. [26] indicates that asprosin results in an impairment of insulin sensitivity in the skeletal muscles. It is known that the experimental dysfunction of asprosin by immunological or genetic methods causes a significant reduction in the concentration of glucose and insulin in the blood [19]. It was also shown that pancreatic β cells are a source of asprosin under hyperlipidemia conditions, and asprosin induces the inflammation, dysfunction, and apoptosis of β cells, resulting in impaired insulin secretion [27]. It has also been demonstrated that asprosin reduces stress-induced oxidative damage and the apoptosis of mesenchymal stromal cells by the upregulation of the expression of the mitochondrial superoxide dismutase [28].

The high prevalence of metabolic syndrome and coexisting diseases emphasizes the social importance of this problem and the need to seek effective therapeutic models, including non-pharmacological methods. One of such methods may be the multidirectional action (hormonal, immunological, antioxidant, metabolic) of whole-body cryotherapy (WBC) [29], involving short-lasting (1–3 min) exposure of the whole body to cryogenic temperatures (from −110 °C to −160 °C). It has been previously shown that the use of 20 WBC treatments in healthy young men improves lipid profiles [30]. Similar results were obtained in obese men, using two series of 20 WBC treatments combined with physical training [31]. The improvement of lipid profile was also obtained in studies by Ziemann et al. [32] and Stanek et al. [33]. In a single study of people with low physical capacity, as a result of 10 WBC treatments, there was an increase in the concentration of irisin levels in the blood [34].

However, to date, no studies have been conducted regarding the effects of WBC treatments on blood levels of asprosin. Previous studies among people with metabolic syndrome regarded the relationship between basic levels of asprosin and other risk factors of metabolic disorders [35].

The aim of our research was to determine whether regular exposure of the whole body to cryogenic temperatures has an effect on changes in glucose, asprosin, and other hormones related to metabolism (leptin, adiponectin, irisin) and insulin resistance in menopausal women with metabolic disorders. At the same time, the objective of our study was to conclude whether there is a relationship between the concentrations of asprosin and glucose as well as between lipid profile and the level of other earlier mentioned hormones, regulating the metabolism of carbohydrates and lipids. The participants were exposed to 20 WBC sessions. We hypothesized that 20 WBC treatments have a positive effect on the metabolism of women during menopause through changes in the concentration of selected adipose tissue hormones and a reduction in the insulin resistance of tissues.

## 2. Materials and Methods

### 2.1. Participants

The study participants included 37 women aged 55–70 during menopause who had not had menstruation for at least 12 months and had not underwent WBC treatments for at least 6 months prior to the study. These were people demonstrating low or moderate physical activity, were non-smokers, with non-specific diets (e.g., vegetarian, vegan, diabetic), or taking dietary supplements, who did not permanently take any medication, and for whom there were no contraindications to undergo WBC [36]. The subjects were asked to retain their previous physical activity and diets (which were controlled) and not to use any biological regeneration treatments while being subjected to WBC.

The group was deliberately selected according to age and gender. Hormonal changes in the menopausal period, as a factor independent of lifestyle (diet, physical activity), adversely affect metabolism, leading to the development of abdominal obesity, low-intensity chronic inflammation, and insulin resistance. Menopausal women are the group most exposed to MetS dysfunction [4,8]. Out of the 79 volunteers, 41 met the inclusion criteria, including four who resigned from the study. Finally, among the participants, the MetS group with metabolic syndrome (*n* = 19) and the control group (CON, *n* = 18) without MetS who completed the entire research program were distinguished.

In the diagnosis of the metabolic syndrome, criteria for women were adopted, as defined by the National Cholesterol Education Program Adult Treatment Panel III (NCEP–ATP III) [1,2,4]. Metabolic syndrome was diagnosed in subjects who met at least three of the following criteria:

1. waist circumference >88 cm
2. triglycerides ≥150 mg/dL
3. high-density lipoprotein (HDL) <50 mg/dL
4. fasting glucose ≥100 mg/dL
5. systolic blood pressure (SBP) ≥130 mmHg or diastolic blood pressure (DBP) ≥85 mmHg or antihypertensive therapy.

In the MetS and CON groups, there were 10 and five women with hyperglycemia, respectively (HG group, *n* = 15), whose fasting blood glucose level was >99 mg/dL (>5.5 mmol/L). The remaining women from both groups were characterized by normoglycemia (NG group, *n* = 22), i.e., fasting glucose 60–99 mg/dL (3.4–5.5 mmol/L) [37].

### 2.2. Study Design

During the first stage, somatic measurements and assessment of body composition were performed. The participants underwent internist, cardiology, and gynecological diagnostics.

During the second stage, a series of 20 WBC treatments over a period of 4 weeks and biochemical determination were conducted.

The study was carried out in accordance with the Declaration of Helsinki. The methodology of the study was approved by the Bioethical Committee of the Regional Medical Chamber (96/KBL/OIL/2015, 03/07/2015). The participants were informed in detail about the purpose and course of the research and about the possibility of resigning from participation at any stage without giving a reason. Having read the written information about the course of the research, the participants provided written consent to participate in the study, as well as the use of personal data and research results for scientific purposes.

### 2.3. Somatic Measurements and Body Composition Assessment

Before beginning WBC, body mass measurements (Jawon IOI-353 Body Composition Analyzer, Gyeongsan, Korea), body height (Seca 217, Hamburg, Germany), and waist and hip circumference (Seca 201, Hamburg, Poland) to the nearest 1 mm were taken in the fasting state. Body composition assessment was performed in fasting state using Dual Energy X-Ray Absorptiometry (DEXA), GE Healthcare Lunar iDXA (GE Medical System, Freiburg, Germany). The examination was carried out in supine position while not moving. The subjects were dressed in a cotton sleeveless top and shorts. They were asked to remove plastic, rubber, and metal objects as well as jewelry.

### 2.4. Whole-Body Cryotherapy Treatments

WBC treatments were performed under the supervision of a physician and physiotherapist in a stationary cryogenic chamber (Bamet KN-1), consisting of a vestibule and a main chamber, equipped with an audiovisual system, allowing the monitoring of treatments and communication with the examined persons. The medium that cooled the air was liquid nitrogen. The temperature inside the chamber was continuously recorded, and the air was dried.

WBC treatments were performed every day in the afternoon (15:00–17:00) in four consecutive series, five treatments per series, excluding Saturdays and Sundays. The dates of treatments were selected so that individuals would participate in subsequent treatments at similar times.

Immediately prior to each WBC session, after 15 min of rest in a seated position, SBP and DBP measurements were performed among the volunteers in thermoneutral conditions. In any case, the measurement results did not exceed 150/90 mmHg, which is the value allowing the treatment to be performed [38].

Each WBC treatment began with a 30-second (s) stay in the vestibule (−60 °C). Then, the participants entered the main chamber, where they stayed at −130 °C for 3 min. Four people participated in the WBC procedure at once. During the procedure, the women moved walking quietly “in a circle”—one after the other, changing the direction of the march every 30 s. After WBC, they entered the room at 20–22 °C and remained there for 20 min.

During the WBC procedure, the women were dressed in sleeveless cotton tops and shorts that did not put pressure on the skin; clothing was free of rigid, including metal and elements. Before undergoing the WBC procedure, the women took off their jewelry and glasses; they did not use contact lenses. In addition, each of the subjects was provided with a surgical mask with a layer of gauze covering the nose and mouth, wool socks protecting the ankle and knee joints, gloves, headbands, or caps covering the ears, and clogs. The study participants were asked not to apply cosmetics on their skin before entering the cryochamber and to remove sweat, the presence of which could cause frostbite.

### 2.5. Biochemical Markers

Biochemical markers were determined in the blood taken before the beginning of the series of WBC treatments (pre-WBC) and in the morning (06:00–07:30) the day following the 20 WBC treatments (after WBC). Venous blood was collected from the elbow joint in a fasting state after about 8 hours of sleep (the last meal was consumed 2 hours before bedtime) using the BD Vacutainer® vacuum system (Becton Dickinson, Franklin Lakes, NJ, USA). Blood samples were sealed prior to the assays using a letter-digit code.

Blood collected for glucose determination (ethylenediaminetetraacetic acid (EDTA) and glycolysis inhibitors: sodium fluoride and potassium oxalate) and asprosin, irisin, leptin, and adiponectin (EDTA and protease inhibitor: aprotinin 0.6 Trypsin Inhibitor Unit/1 mL of blood) was centrifuged (relative centrifugal force 1.000×g) directly after collection for 15 minutes at 4 °C using MPW-351R centrifege (MPW Med. Instruments, Warsaw, Poland). For the assay of insulin, test tubes with a clotting activator were used; after the collection of blood, these were stored for 20 min at 20–22 °C until a clot was obtained, and then they were centrifuged under the above conditions. The obtained plasma and serum were stored until analyzed at −70 °C (ultra-low temperature freezer POL-EKO-APARATURA ZLN-UT 300 PREM, Wodzislaw Slaski, Poland).

Glucose concentration (enzymatic method) and insulin (electrochemiluminescence) were determined in accordance with the manufacturer’s manual using reagents dedicated to the Cobas c 701/702 biochemical analyzer and Cobas e 801, respectively (Roche Diagnostics International Ltd., Rotkreuz, Switzerland). The test range for glucose (GLUC3) was 2–750 mg/dL, while for insulin (Elecsys Insulin), it was 0.4–1000 mU/mL. The insulin resistance index was calculated according to Equation (1):

HOMA-IR = insulin (mU/mL) × glucose (mmol/L)/22.5(1)

The concentrations of asprosin and irisin were determined using the SK00229-09 (Aviscera Bioscience, Inc., Santa Clara, CA, USA) and EK-067-16 (Phoenix Pharmaceuticals, Inc., Burlingame, CA, USA) tests, respectively. The concentration of leptin was determined using the Human Leptin Elisa RD191001100 test, whereas adiponectin concentration was determined with the Human Adiponectin Elisa RD191023100 high-sensitivity test (BioVendor, Karasek, Czech Republic). Plasma for the determination of adiponectin concentrations was diluted 300-fold. Determinations were performed using the immunoenzymatic method (ELISA) with the measurement of absorbance with E-LizaMat 3000 microplate reader, (DRG International, Inc., Springfield, NJ, USA) according to the methodology presented by the manufacturer, reading the results from the standard curve performed during each test.

The detection range for asprosin was 1–32 nmol/L; for irisin, it was 0–100 ng/mL; for leptin, it was 0–50 ng/mL; and for adiponectin, it was 2–150 ng/mL. The intra-assay coefficient of variation (CV) was 4.4% for adiponectin and 7.6% for leptin, and the inter-assay CV for adiponectin was 6.2%, while for leptin, this value totaled 6.7%.

### 2.6. Assessment of Physical Activity and Nutrition

The physical activity (PA) of the subjects was assessed using the International Physical Activity Questionnaire (IPAQ), Polish version [39] before the treatment and during the fourth week of WBC application. Prior to the study and during the second and fourth weeks of using WBC, volunteers were asked to register meals with 7-day diaries. On the basis of the declared menus, using the computer program Dieta 5.0 (Food and Nutrition Institute, Warsaw, Poland), the nutritional method was evaluated in terms of quantity and quality during the period of WBC application. Serving sizes were determined based on a photo album of products and dishes [40].

### 2.7. Statistical Analysis

The distribution of results for the analyzed variables was checked using the Shapiro–Wilk test. The significance of differences between groups in the case of individual measurements was assessed using tests for independent samples (Student’s *t*-test or the Mann–Whitney U non-parametric test). Analysis of variance with repeated measurements (ANOVA) was used to compare the influence of WBC treatments on changes in the analyzed variables in the compared MetS and control (CON) groups as well as the HG and normoglycemic (NG) groups. In the case of a significant influence of any of the main factors, i.e., GROUP, WBC, and GROUP × WBC interactions, the significance of differences between specific averages was checked by performing a statistical analysis for planned comparisons: the Fisher test, based on the Student’s *t* test (post-hoc). The influence of WBC treatments on biochemical indicators in the whole group (*n* = 37) was also assessed. The Student’s *t*-test or non-parametric Wilcoxon test were used. The statistical significance of differences between the averages compared was assumed at the level of *p* < 0.05. The STATISTICA 12 package (StatSoft, Inc., Tulsa, OK, USA) was used.

## 3. Results

### 3.1. Characteristics of the Participants

The age of the subjects in the compared groups was similar (*p* > 0.05). People in the MetS group, compared to CON, had significantly higher body fat (FAT) and lean body mass (LBM), which resulted in a greater overall body mass (*p* < 0.05). In the MetS group, BMI, FAT content in total body mass, and the visceral fat/hip ratio (*p* < 0.05) were also higher compared to CON. In the HG and NG groups, no differences in body mass or composition were found (Table 1).

Among the diagnostic criteria of metabolic syndrome in the MetS group, compared to CON, a higher mean level of glycemia (*p* < 0.05) and lower HDL concentration (*p* < 0.05) and a larger waist circumference (*p* < 0.05) were found (Table 2). The mean values of TG, SBP, and DBP did not differ (*p* > 0.05) in the MetS and CON groups. In the range of these indicators in the HG group, only glucose concentration was significantly higher (*p* < 0.05) compared to NG (Table 2).

The higher number of leukocytes (*p* < 0.05) and a smaller percentage of monocytes (*p* < 0.05) in the total leukocytes, as well as a higher percentage of glycosylated hemoglobin (*p* < 0.05) and higher level of atherogenic index of plasma (AIP) in the MetS group (*p* < 0.05), and a higher concentration of CRP (*p* < 0.05) compared to CON were found (Table 3). In the HG group, the percentage of glycated hemoglobin was higher than in the NG group (*p* < 0.05). The HG and NG groups did not differ in terms of blood count, AIP, or CRP (*p* > 0.05) (Table 3).

None of the patients were diagnosed with chronic conditions or contraindications for the use of WBC treatments [37].

### 3.2. Biochemical Indicators

The numerical values of the analyzed variables and the results of statistical analysis in the MetS and CON groups, in the HG and NG groups, as well as in the whole group (*n* = 37) are shown in Table 4.

#### 3.2.1. Glucose

In the MetS and CON groups, significant differences between groups (GROUP factor) were noted in glucose concentration (*F* = 5.58, *p* = 0.02), while neither the WBC nor GROUP×WBC influenced glucose concentration in these groups (ANOVA). When comparing the results between the HG and NG groups, significant group-related differences (GROUP factor) were found in glucose concentration (*F* = 6.52, *p* < 0.01), as well as a significant WBC effect regarding glucose concentration changes (*F* = 6.06, *p* = 0.02), while the changes in glucose concentration following WBC differed (*F* = 6.90, *p* = 0.01) in the HG and NG groups (GROUP×WBC) (ANOVA).

**Figure 1:**
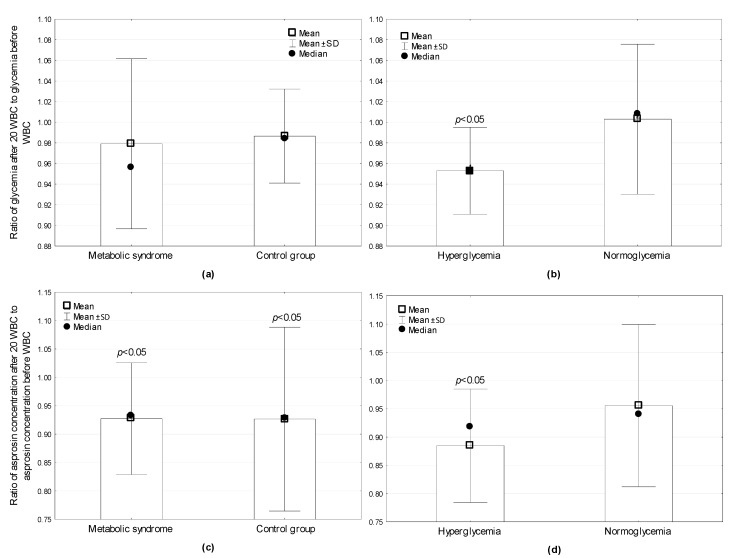
Comparison of changes in plasma glucose and asprosin concentration after 20 whole body cryotherapy treatments in the studied groups: (**a**) change in glucose concentration in the group with metabolic syndrome compared to the control group; (**b**) change in glucose in the hyperglycemic group compared to the normoglycemic group; (**c**) change in asprosin concentration in the group with metabolic syndrome compared to the control group; (**d**) change in asprosin concentration in the hyperglycemic group compared to the normoglycemia group; *p* < 0.05: statistically significant changes.

Post-hoc analysis showed that glucose concentration before WBC was significantly higher in the MetS and HG groups compared to CON (*p* = 0.022) and NG (*p* < 0.001). After WBC, the glucose concentration significantly decreased (*p* = 0.002) only in the HG group (Figure 1); however, its value was still higher (*p* < 0.001) than in the NG group. Analyzing the results for the whole group (*n* = 37), a significant reduction in glucose concentration (*p* = 0.04) was noted after WBC.

#### 3.2.2. Insulin

Analyzing the results in the MetS and CON as well as in HG and NG groups (ANOVA), the only significant differences between groups (GROUP factor) regarding insulin concentration (*F* = 8.94, *p* = 0.01) were found between the MetS and CON groups. The insulin concentration before and after WBC was significantly higher (post-hoc) in the MetS compared to the CON group (*p* = 0.009).

#### 3.2.3. HOMA-IR

Significant differences between groups (GROUP factor) were found in the HOMA-IR values in the MetS and CON groups (*F* = 10.37, *p* < 0.01), as well as in the HG and NG groups (*F* = 5.35, *p* = 0.03) (ANOVA). Post-hoc analysis showed that the HOMA-IR value before WBC was significantly higher in the MetS and HG groups, compared to CON (*p* = 0.005) and NG (*p* = 0.007), respectively. After the WBC sessions, the HOMA-IR value was higher in the MetS group than in the CON group (*p* = 0.007).

#### 3.2.4. Asprosin

Significant group-related differences (ANOVA, GROUP factor) in asprosin concentration were found only in the HG and NG groups (*F* = 4.56, *p* = 0.04), whereas a significant effect of WBC factor on asprosin concentration was found by comparing the results in the MetS and CON groups (*F* = 7.19, *p* = 0.01) and in the HG and NG groups (*F* = 10.18, *p* < 0.01) (ANOVA). Differences between groups regarding changes in asprosin concentration after WBC (GROUP×WBC) were found by comparing the results in the HG and NG groups (*F* = 4.35, *p* = 0.04). Post-hoc analysis showed that the concentration of asprosin before (*p* = 0.023) and after WBC (*p* = 0.046) was higher in the HG compared to the NG group. After WBC, the concentration of asprosin decreased compared to the baseline value in the MetS (*p* = 0.003), CON (*p* = 0.025), and HG (*p* = 0.002) groups (Figure 1), and in the whole group (*p* < 0.001).

#### 3.2.5. Irisin

Significant differences between groups (ANOVA, GROUP factor) were found in the concentration of irisin in the HG and NG groups (*F* = 5.09, *p* = 0.03). The irisin concentration before WBC was significantly lower in the HG group compared to the NG group (post-hoc, *p* = 0.038). Irisin concentration did not change after WBC in any of the groups (*p* > 0.05).

#### 3.2.6. Leptin

Significant group-related differences (ANOVA, group factor) in leptin concentrations were found in the MetS and CON groups (*F* = 6.43, *p* = 0.02). The leptin concentration before (*p* = 0.025) and after WBC (*p* = 0.013) was significantly higher in the MetS group compared to the CON (post-hoc). The concentration of leptin did not change after WBC in any of the groups (*p* > 0.05).

#### 3.2.7. Adiponectin

Significant differences between groups (ANOVA, GROUP factor) regarding adiponectin concentration in the HG and NG groups were found (*F* = 4.97, *p* = 0.03). The adiponectin concentration before (*p* = 0.043) and after WBC (*p* = 0.027) was significantly lower in the HG compared to the NG group (post-hoc). WBC treatments did not affect the change in adiponectin concentration among the groups under study (*p* > 0.05).

#### 3.2.8. Leptin/Adiponectin Indicators

Significant (*F* = 11.38, *p* < 0.01) group-related differences (ANOVA, GROUP factor) in leptin/adiponectin index (Lept/Adipo) values were found. The value of the Lept/Adipo index in the MetS group was significantly higher than in the CON group (post-hoc, *p* = 0.002) both before and after WBC procedures. In none of the groups did the WBC treatments have a significant impact on Lept/Adipo changes (*p* > 0.05).

### 3.3. Correlations

A significant (*p* < 0.05) positive correlation was found between the baseline concentration of asprosin and glucose (*r* = 0.55) (Table 5), as well as a significant (*p* < 0.05) positive correlation between the changes in asprosin and glucose concentrations as a result of 20 WBC treatments (*r* = 0.36) in all of the examined women.

In addition, a significant positive correlation was found between the baseline concentration of asprosin and the ratio of the Lept/Adipo concentration (*r* = 0.59), TG concentration (*r* = 0.62), and atherogenic index of plasma (AIP, *r* = 0.41) before the WBC procedures. At the same time, the change in asprosin concentration after 20 WBC sessions negatively correlated (*p* < 0.05) with the baseline level of these variables, i.e., with Lept/Adipo (*r* = −0.48), TG (*r* = −0.55), and AIP (*r* = −0.37), respectively. There were no statistically significant correlations between the baseline concentration of asprosin or between the changes in asprosin concentration and the baseline level of insulin, HOMA-IR, irisin, leptin, adiponectin, HDL, glycated hemoglobin (HbA_1c_,) and CRP (Table 5). There were no correlations between asprosin concentration, body mass, and BMI.

## 4. Discussion

Our research was the first to show that the repeated action of cryogenic temperatures on the whole body affects the reduction of blood levels of asprosin in menopausal women, regardless of whether they have MetS. After applying 20 WBC treatments, we found that asprosin levels decreased in both women with MetS and in the CON group. Comparing the results obtained for women with hyperglycemia (>5.5 mmol/L) to the results of subjects with normal glucose concentrations, we found a decrease in asprosin level in the blood after WBC only in the HG group, although we did not note a significant relationship between the change in asprosin concentration after WBC and the baseline concentration of glucose in the blood.

Furthermore, we found that in women with hyperglycemia, in addition to lowering the blood levels of asprosin, WBC also reduces the blood glucose measured in the fasting state. At the same time, we noted that the reduction in glucose levels after 20 WBC treatments significantly correlates with the decrease of asprosin in the blood levels. However, in none of the groups did we find changes in the concentration of other adipocytokines or the insulin resistance index as a result of WBC.

In our study, we showed that the concentration of asprosin in the blood before beginning WBC was positively (statistically significant) correlated with the level of glucose in the blood. Just as stated by Wang et al. [24], women with hyperglycemia had a higher concentration of asprosin than women with normal glucose levels. However, we did not find any significant differences in the level of asprosin in women with MetS compared to the CON group, which was a convergent result of previous research by Chang et al. [35]. Our findings show that an important factor related to the level of asprosin is the elevated level of glucose in the blood, which was not an obligatory condition in the diagnostics of MetS. The concentration of asprosin was positively correlated with the ratio of leptin to adiponectin concentration, which was significantly higher in the MetS group compared to CON, while it did not differ depending on the glucose level in the studied groups. We also found a positive relationship between the circulating asprosin concentration and the atherogenesis rate and TG, the level of which is disturbed in the case of metabolic syndrome and insulin resistance. The reduction in blood levels of asprosin after 20 WBC treatments was independent of baseline glucose levels, while it was negatively correlated with baseline levels of TG, AIP, and the leptin/adiponectin ratio. We did not find any relationships between the baseline concentration of asprosin in the blood, its changes after WBC, and the concentrations of irisin, leptin, and adiponectin. In addition, insulin levels, HOMA-IR, HbA1c, and CRP were not related to the concentration of asprosin or its changes after WBC.

In the study by Li et al. [41], among 66 healthy women and 53 patients with T2DM and 41 patients with PCOS, the plasma concentration of asprosin in those with T2DM and PCOS was comparable and was higher in both groups than in healthy women. In both groups of patients, in contrast to our results, plasma levels of asprosin positively correlated with HOMA-IR and HbA1c. Similar results were obtained in another group of patients with T2DM [28], indicating a higher level of asprosin in the plasma of patients and in comparison to normoglycemia, and a positive correlation was found between the level of this hormone and fasting glucose and HOMA-IR, although similarly as in our research, there was no correlation between asprosin and HbA1c. In some studies, there was a positive correlation between the concentration of asprosin, BMI, and WHR [28,41] as well as SBP and DBP [41]. Conducting research among 116 volunteers who, according to BMI value, were assigned to low weight (<18.5), normal weight (18.5–24.9), overweight (25.0–29.9), obese class I (30.0–34.9), obese class II (35.0–39.9), and obese class III (≥40.0) groups, serum asprosin concentrations have been shown to increase gradually with increasing BMI [42]. The same research also showed that the interlobular striated ducts and the interlobular ducts of the submandibular and parotid salivary glands produce asprosin, the concentration of which in saliva is correlated with the serum concentration, and which also increases along with the increase in BMI [42]. Asprosin in the saliva, similar to serum, was positively correlated with glucose, total cholesterol, and LDL, and negatively with HDL. The relationship between asprosin concentration and BMI was not confirmed by Chang et al. [35]. In our study, we did not find a relationship between plasma asprosin concentration, BMI, and body mass in elderly women with metabolic disorders, as earlier indicated by Wiecek et al. [43] in young healthy people. Similarly as in the case of young people [43], in the research by Schumann et al. [44], there was no gender-related difference in the level of asprosin in the group of people around the age of 50. In addition, in the elderly, the concentration of this hormone in the blood was similar in obese and non-obese individuals [44]. However, in obese children, the concentration of asprosin in the blood was lower than in children with normal body weight, and at the same time, it was lower in boys than in girls [25]. In the research by Chang et al. [35], the concentration of asprosin in the group meeting MetS criteria was alike that in the control group, similarly as in our study. However, the MetS group was characterized by a higher concentration of irisin [35], which was not confirmed in our research. In our study, the higher concentration of irisin was characteristic of people with normoglycemia compared to those with hyperglycemia.

Maintaining isothermia in conditions of whole-body exposure at −130 °C requires an intensification of non-shivering thermogenesis in brown adipocytes (BAT). According to a review of the research, exposure to cold increases the activity of mitochondrial enzymes and oxygen consumption, and at the same time, increases the expression of UCP-1, which is the reason for the uncoupling of oxidative phosphorylation and increased energy dissipation [45]. The main source of energy for non-shivering thermogenesis is the oxidation of glucose and free (non-esterified) fatty acids (FFA), the uptake of which in BAT is increased under the influence of cold, indirectly as a result of the action of released norepinephrine (NE) [46]. It has been shown in animal models that exposure to lowered temperatures corrects hyperlipidemia by NE acting on the lipolysis. NE also activates gene expression for vascular endothelial growth factor (VEGF), which increases capillary permeability and affects the activity of lipoprotein lipase (LPL), releasing FFA from triglyceride-rich lipoproteins (chylomicrons). FFA uptake by BAT cells is facilitated by the CD36 transmembrane receptor [46,47]. The blood concentration of NE showed a significant two to threefold increase after long-term treatment of WBC (−110 °C for 2 min, three times for 12 weeks), which was used in healthy women [48].

In our research, we decided to use 20 WBC treatments, applied once a day in four series of five treatments, due to the beneficial metabolic effects of this number of treatments affecting the change of the blood lipid profile observed by Lubkowska et al. [30]. In the study by Lubkowska et al. [30,31], after 20 WBC sessions, there was a decrease in total cholesterol, TG, LDL, and an increase in HDL concentration in healthy as well as obese men. In other studies, after 10 WBC, but applied twice a day for five consecutive days, LDL cholesterol, LDL/HDL, and TC/HDL were reduced [32]. Lipid metabolism and the level of lipid metabolites in the blood are associated with the concentration of adipocytokines [49]. However, we did not achieve the expected increase in adiponectin and irisin levels or a decrease in blood leptin concentration as a result of WBC treatments in women during menopause. Also, in obese men, WBC (1, 5, 10, and 20 treatments) did not affect the level of adiponectin or leptin in blood [31,50], while in contrast to our research, in the study by Dulian et al. [34], as little as 10 WBC treatments caused increased blood levels of irisin in obese middle-aged men.

As demonstrated in one of the recent studies by Lubkowska et al. [51], beneficial changes in lipid metabolism as a result of whole-body cryotherapy require the use of at least 20 exposure sessions to cryogenic temperatures. The increase in the number of treatments to 30 was one reason for the further decrease in TC and TG concentrations, and at the same time, an increase in HDL concentration in the blood. These results [50] may indirectly indicate the activation of BAT under the influence of WBC treatments. In the research by Lubkowska et al. [51], in contrast to our studies, participants were young non-obese men, and lipid metabolism was determined on the basis of changes in blood lipid profile, while adipocytokines and the molecular mechanism of the obtained changes were not determined. Our research involved older women with increased body fat, in whom both the content and BAT activity were lower than in young non-obese individuals [52]. Presumably, the reason for the lack of changes in adipocytokine levels in our studies is the insufficient number of applied WBC treatments. In such a group, in order to obtain beneficial changes in the adipocytokine profile and to normalize lipid metabolism, it seems justified to perform at least 30 or more WBC procedures.

In our research, we also evaluated the effect of cryogenic temperatures on glucose, insulin, and HOMA-IR. After applying 20 WBC treatments, no significant differences in insulin concentration or insulin resistance index were found in any of the groups under study. Only in the hyperglycemic group was there a reduction in blood glucose level. In other studies, it was found that the use of WBC 20 series did not affect the level of glycemia in obese or healthy men [31,53].

Repeated exposure to cold temperatures, regardless of insulin level, increases glucose uptake by increasing the type 4 glucose transporter (GLUT4) expression in BAT, which was demonstrated in an animal model [54]. Glucose uptake in BAT among obese, glucose-intolerant mice was higher than in lean mice under cold conditions [47]. In human studies, it was found that BAT glucose uptake activated by cold exposure correlates with the normalization of blood glucose levels, and at the same time, is greater than glucose uptake stimulated by insulin [55]. The results of the above studies [47,54,55] indicate the up-regulation of GLUT4 expression as a result of WBC as a potential mechanism responsible for the lower blood glucose levels found in our own research conducted among women with hyperglycemia, despite no changes in insulin levels. Wang et al. [24] showed that the increase in blood insulin concentration stimulated in the first phase after glucose administration does not significantly affect the concentration of asprosin, which indicates the independent secretion of these hormones. Also in our research, the decrease in blood levels of asprosin after WBC was not associated with changes in insulin levels. Perhaps the decrease in asprosin concentration under the influence of WBC observed in our study is associated with changes in the secretion of other pro-inflammatory cytokines such as visfatin and resistin, the blood levels of which, despite no changes in leptin and adiponectin levels, decreased after 10 WBC procedures in obese men with low physical activity [50].

So far, only the impact of physical exercise on changes in asprosin secretion has been studied. It has been shown that as a result of a single anaerobic effort, the concentration of asprosin in the blood of young, non-obese individuals increases [43], while increasing the intensity does not change the concentration of this hormone in the blood of obese people or those with correct body composition at the age of about 50 [44]. Recent studies on rats have shown that interval and/or aerobic training is an effective method of reducing asprosin levels in metabolic syndrome [56] and type 1 diabetes (T1DM) [57], respectively.

Our study has shown a significant decrease in the concentration of asprosin in the blood after 20 WBC treatments in the group with hyperglycemia, as well as in women with diagnosed metabolic syndrome and in the control group. The asprosin concentration did not decrease as a result of WBC only in the normoglycemia group, regardless of the presence of MetS. This indicates a significant effect of carbohydrate disorders on asprosin secretion. These are the first studies on this topic. Considering the previously demonstrated relationship between asprosin and insulin resistance [23], our research indicates the potentially beneficial effects of WBC treatments in supporting metabolic disorders, T2DM, and insulin resistance.

A limitation of our study is the small number of participants and the lack of evaluation of pro- and anti-inflammatory interleukins and redox balance indicators. Subsequent research should focus on the evaluation of mRNA expression for adipocytokines, including asprosin for both sexes, in combination with anti-inflammatory and antioxidant effects as a result of repeated exposure to cryogenic temperatures in people differing in body composition. Research should be conducted applying at least 30 WBC treatment sessions.

## 5. Conclusions

In menopausal women, there is a statistically significant positive correlation between the concentration of asprosin in the blood and the level of risk factors for metabolic disorders such as fasting glucose, atherogenesis index and leptin/adiponectin ratio. The concentration of asprosin in the blood is reduced as a result of 20 WBC treatments in menopausal women regardless of the prevalence of metabolic syndrome. Research indicates the possibility of using WBC treatments in supporting metabolic disorders, T2DM, and insulin resistance.

## Figures and Tables

**Table 1:** Age and body composition in the groups under study.

Variable	MetS (*n* = 19)	CON (*n* = 18)	HG (*n* = 15)	NG (*n* = 22)	ALL (*n* = 37)
Age (years)	61.53 ± 3.99	60.28 ± 3.63	60.47 ± 3.38	62.00 (56.00–64.00)	62.00 (58.00–64.00)
Body mass (kg)	77.36 ± 11.95 *	66.32 ± 6.23	72.87 ± 12.76	68.25 (65.90–74.10)	69.60 (65.90–76.60)
BMI (kg/m^2^)	30.09 ± 4.98 *	25.5 ± 2.37	27.74 ± 5.20	26.58 (25.15–28.96)	26.60 (25.15–29.23)
LBM (kg)	44.19 ± 4.75 *	40.76 ± 3.31	43.86 ± 5.22	42.00 ± 3.81	42.52 ± 4.41
FAT (kg)	33.17 ± 8.04 *	25.56 ± 4.08	30.57 ± 9.95	29.76 ± 67.18	28.14 (25.43–32.68)
FAT (%)	42.42 ± 4.32 *	38.38 ± 3.49	39.99 ± 4.59	40.76 ± 4.32	40.45 ± 4.39
FAT visceral (%)	48.98 ± 5.94 *	42.74 ± 5.58	46.05 ± 6.42	45.87 ± 6.71	45.95 ± 6.51
FAT hip region (%)	44.24 ± 4.66	41.60 ± 3.40	42.03 ± 4.91	43.59 ± 3.73	42.95 ± 4.26
FAT visceral/hip	1.11 ± 0.10 *	1.03 ± 0.11	1.10 ± 0.12	1.05 ± 0.10	1.07 ± 0.11
WHR	1.00 ± 0.02	1.01 ± 0.01	1.00 ± 0.02	1.01 ± 0.01	1.01 ± 0.02

Values are means ± SD or median (IQR); SD: standard deviation, IQR: interquartile range; MetS: group with metabolic syndrome, CON: control group; HG: hyperglycemia, NG: normoglycemia; * *p* < 0.05, significant differences MetS vs. CON or HG vs. NG (Student’s *t* test or Mann–Whitney U test); BMI: body mass index, LBM: lean body mass, FAT: body fat, WHR: waist–hip ratio.

**Table 2:** Value of variables comprising diagnostic criteria of metabolic syndrome according to National Cholesterol Education Program Adult Treatment Panel III (NCEP–ATP III) in the compared groups.

Variable	MetS (*n* = 19)	CON (*n* = 18)	HG (*n* = 15)	NG (*n* = 22)	ALL (*n* = 37)
Waist circumference (cm)	94.62 ± 9.50 *	82.52 ± 8.48	90.29 ± 13.49	87.71 ± 8.70	88.78 ± 10.79
TG (mg/dL)	130.65 ± 41.40	111.47 ± 38.47	120.46 ± 28.81	103.69 (84.88–166.25)	112.00 (87.50–147.00)
HDL (mg/dL)	51.47 * (47.60–58.82)	60.95 (56.50–73.53)	56.50 (50.31–65.79)	57.08 (51.47–63.08)	56.50 (51.08–63.08)
Glucose (mg/dL)	102.39 ± 9.81 *	92.93 ± 6.94	103.50 * (100.80–106.02)	94.71 ± 9.31	97.79 ± 9.69
SBP (mmHg)	127.63 ± 17.27	120.28 ± 16.84	125.33 ± 19.86	123.10 ± 15.59	124.00 ± 17.22
DBP (mmHg)	82.63 ± 7.88	77.78 ± 7.71	82.33 ± 7.53	78.91 ± 8.28	80.30 ± 8.06

Values are means ± SD or median (IQR); SD: standard deviation, IQR: interquartile range; MetS: group with metabolic syndrome, CON: control group; HG: hyperglycemia, NG: normoglycemia; * *p* < 0.05, significant differences MetS vs. CON or HG vs. NG (Student’s *t* test or Mann–Whitney U test); TG: triglycerides, HDL: high-density lipoproteins, SBP: systolic blood pressure, DBP: diastolic blood pressure.

**Table 3:** Results of laboratory blood analysis in the groups under study.

Variable	MetS (*n* = 19)	CON (*n* = 18)	HG (*n* = 15)	NG (*n* = 22)	ALL (*n* = 37)
Erythrocytes (mln/µL)	4.62 ± 0.22	4.59 (4.40–4.70)	4.51 ± 0.14 *	4.67 ± 0.25	4.61 ± 0.23
Hemoglobin (g/dL)	13.89 ± 0.55	13.94 ± 0.55	13.96 ± 0.43	13.75 (13.30–14.30)	13.92 ± 0.55
Hematocrit (%)	40.30 (39.30–42.40)	41.14 ± 1.62	40.77 ± 1.37	41.12 ± 1.87	40.60 (39.60–42.10)
ESR (mm/h)	19.58 ± 10.67	16.11 ± 9.65	19.07 ± 9.27	17.09 ± 10.92	17.89 ± 10.19
Platelets (tys/µL)	246.53 ± 77.69	254.89 ± 56.59	242.13 ± 76.12	245.50 (222.0–278.0)	250.59 ± 67.44
Leukocytes (tys/µL)	6.16 ± 1.25 *	5.29 ± 1.06	6.01 ± 1.26	5.55 ± 1.20	5.74 ± 1.23
Neutrophils (%)	49.31 ± 8.08	48.36 ± 7.10	51.04 ± 7.06	47.35 ± 7.63	48.85 ± 7.53
Lymphocytes (%)	38.68 ± 8.14	37.56 ± 6.60	36.47 ± 7.26	39.27 ± 7.36	38.14 ± 7.35
Monocytes (%)	8.12 ± 1.57 *	9.00 (8.00–11.70)	8.67 ± 2.10	9.00 ± 1.91	8.87 ± 1.97
Eosinophils (%)	3.00 (2.00–4.00)	3.00 (2.30–4.50)	3.02 ± 1.68	3.00 (2.30–4.40)	3.00 (2.20–4.00)
Basophils (%)	0.90 (0.20–1.00)	0.84 ± 0.59	0.59 ± 0.44	1.00 (0.60–1.00)	0.80 (0.40–1.00)
HbA_1c_ (%)	5.84 ± 0.28 *	5.60 (5.50–5.90)	5.88 ± 0.30 *	5.55 (5.50–5.90)	5.75 ± 0.29
AIP (log_10_TG/HDL)	0.38 ± 0.23 *	0.22 ± 0.20	0.35 ± 0.25	0.27 ± 0.21	0.30 ± 0.23
CRP (mg/L)	1.74 * (0.92–3.64)	0.92 (0.48–1.82)	2.47 ± 3.88	1.45 (0.61–3.07)	1.48 (0.61–2.37)

Values are means ± SD or median (IQR); SD: standard deviation, IQR: interquartile range; MetS: group with metabolic syndrome, CON: control group; HG: hyperglycemia, NG: normoglycemia; * *p* < 0.05, significant differences MetS vs. CON or HG vs. NG (*t*-test or Mann–Whitney U test); ESR: erythrocyte sedimentation rate, HbA_1c_: glycated hemoglobin, AIP: atherogenic index of plasma, TG: triglycerides, HDL: high density lipoproteins, CRP: C-reactive protein.

**Table 4:** Comparison of changes in concentration of glucose, selected hormones, and insulin resistance index (HOMA-IR) under the influence of whole-body cryotherapy (WBC) treatments.

Variable	Time	MetS (*n* = 19)	CON (*n* = 28)		*F(p)*		HG (*n* = 15)	NG (*n* = 22)		*F(p)*		TOTAL (*n* = 37)
				GROUP	WBC	GROUP×WBC			GROUP	WBC	GROUP×WBC	
Glucose	pre WBC	5.56 ± 0.50 *	5.22 ± 0.43	5.58	3.32	0.25	5.89 ± 0.26 *	5.05 ± 0.27	6.52	6.06	6.90	5.39 ± 0.50
(mmol/L)	after WBC	5.42 ± 0.44	5.14 ± 0.39	(0.02)	(0.08)	(0.62)	5.61 ± 0.27 *^#^	5.06 ± 0.38	(<0.01)	(0.02)	(0.01)	5.28 ± 0.43 ^#^
Insulin	pre WBC	12.12 ± 5.65 *	8.28 ± 2.50	8.94	0.28	0.00	12.07 ± 6.15	9.01 ± 3.13	2.54	0.60	1.67	10.25 ± 4.77
(µIU/mL)	after WBC	11.79 ± 5.28 *	7.94±2.89	(0.01)	(0.60)	(0.99)	10.78 ± 4.84	9.33 ± 4.54	(0.12)	(0.44)	(0.21)	9.92 ± 4.65
HOMA-IR	pre WBC	3.03 ± 1.52 *	1.92±0.61	10.37	0.51	0.02	3.16 ± 1.60 *	2.04 ± 0.78	5.35	1.10	2.77	2.49 ± 1.29
	after WBC	2.89 ± 1.41 *	1.83±0.71	(<0.01)	(0.48)	(0.88)	2.71 ± 1.27	2.14 ± 1.18	(0.03)	(0.30)	(0.11)	2.37 ± 1.23
Asprosin	pre WBC	5.35 ± 6.46	4.66 ± 2.13	0.17	7.19	0.27	6.70 ± 7.19 *	3.66 ± 0.64	4.56	10.18	4.35	5.01 ± 4.81
(nmol/L)	after WBC	4.77 ± 5.17 ^#^	4.26 ± 2.05 ^#^	(0.68)	(0.01)	(0.61)	6.07 ± 5.89 *^#^	3.47 ± 0.56	(0.04)	(<0.01)	(0.04)	4.52 ± 3.93 ^#^
Irisin	pre WBC	1.87 ± 0.24	1.89 ± 0.22	0.29	2.14	0.09	1.80 ± 0.25 *	1.94 ± 0.19	5.09	2.30	0.19	1.88 ± 0.23
(µg/mL)	after WBC	1.92 ± 0.19	1.96 ± 0.21	(0.60)	(0.15)	(0.77)	1.87 ± 0.22	1.98 ± 0.18	(0.03)	(0.14)	(0.66)	1.94 ± 0.20
Leptin	pre WBC	19.21 ± 7.25 *	14.09 ± 5.42	6.43	2.02	0.37	15.45 ± 8.08	17.58 ± 5.91	0.68	1.71	0.14	16.72 ± 6.85
(ng/mL)	after WBC	18.80 ± 6.99 *	13.07 ± 6.86	(0.02)	(0.16)	0.55	14.97 ± 8.27	16.73 ± 6.89	(0.41)	(0.20)	(0.72)	16.01 ± 7.42
Adiponectin	pre WBC	8.38 ± 3.06	10.82 ± 4.88	3.77	3.52	2.56	7.77 ± 2.85 *	10.79 ± 4.54	4.97	0.00	0.32	9.56 ± 4.18
(µg/mL)	after WBC	8.26 ± 2.94	11.02 ± 6.13	(0.06)	(0.07)	(0.12)	7.62 ± 3.07 *	10.95 ± 5.50	(0.03)	(0.97)	(0.57)	9.60 ± 4.90
Lept/Adipo	pre WBC	2.56 ± 1.47 *	1.40 ± 0.56	11.38	0.89	0.02	2.29 ± 1.65	1.79 ± 0.88	1.66	0.86	0.00	2.00 ± 1.25
	after WBC	2.47 ± 1.20 *	1.34 ± 0.73	(<0.01)	(0.35)	(0.88)	2.22 ± 1.42	1.72 ± 0.88	(0.21)	(0.36)	(0.98)	1.92 ± 1.14

Data presented as mean ± standard deviation; MetS: group with metabolic syndrome, CON: control group; HG: hyperglycemia, NG: normoglycemia; * statistically significant differences between groups (*p* < 0.05); ^#^ significant influence of WBC (*p* < 0.05); Lept/Adipo: ratio of leptin to adiponectin concentrations, HOMA-IR: homeostasis model assessment of insulin resistance.

**Table 5:** Correlations between plasma asprosin and metabolic risk factors in all subjects.

Variable	Plasma Asprosin Level	
pre WBC	pre WBC	Change after 20 WBC
Glucose	0.55 *	NS
Insulin	NS	NS
HOMA-IR	NS	NS
Irisin	NS	NS
Leptin	NS	NS
Adiponectin	NS	NS
Leptin/Adiponectin	0.59 *	−0.48 *
TG	0.62 *	−0.55 *
HDL	NS	NS
AIP	0.41 *	−0.37 *
HbA_1c_	NS	NS
CRP	NS	NS

* Statistically significant correlation (*p* < 0.05): Pearson’s correlation coefficient, NS: non-significant correlation (*p* > 0.05); HOMA-IR: insulin resistance index, TG: triglycerides, HDL: high density lipoproteins, AIP: atherogenic index of plasma (log_10_TG/HDL), HbA_1c_: glycated hemoglobin, CRP: C-reactive protein, WBC: whole body cryotherapy.
